# Serum *N*-glycome alterations in colorectal cancer associate with survival

**DOI:** 10.18632/oncotarget.25753

**Published:** 2018-07-17

**Authors:** Stefan W. de Vroome, Stephanie Holst, Mar Rodriguez Girondo, Yuri E.M. van der Burgt, Wilma E. Mesker, Rob A.E.M. Tollenaar, Manfred Wuhrer

**Affiliations:** ^1^ Department of Surgery, Leiden University Medical Center, 2300 RC Leiden, The Netherlands; ^2^ Center for Proteomics and Metabolomics, Leiden University Medical Center, 2300 RC Leiden, The Netherlands; ^3^ Department of Medical Statistics and Bioinformatics, Leiden University Medical Center, 2300 RC Leiden, The Netherlands

**Keywords:** N-glycans, colorectal cancer, serum biomarker panel, prognosis, survival

## Abstract

Proteins are routinely measured in clinical laboratories for diagnosis, prognosis and therapy monitoring. Nevertheless, both test improvements (performance) and innovations (biomarkers) are needed, and protein *N*-glycosylation offers a rich source of potential markers. Here, we have analyzed the total serum *N*-glycome in a matched case-control study (124 cases versus 124 controls) of colorectal cancer patients. The results were validated in an independent sample cohort (both 61 cases versus 61 controls) and further tested in post-operative samples of cured patients. Our results revealed significant differences between patients and controls, with increased size (antennae) and sialylation of the *N*-glycans in the colorectal cancer patient sera as compared to mainly di-antennary *N*-glycans in sera from controls. Furthermore, glycan alterations showed strong associations with cancer stage and survival: The five-year survival rate largely varied between patients with an altered serum N-glycome (46%) and an *N*-glycome similar to controls (87%). Importantly, the total serum *N*-glycome showed prognostic value beyond age and stage. This clinical glycomics study provides novel serum biomarker candidates and shows the potential of total serum *N*-glycans as a prognostic panel. Moreover, serum N-glycome changes reverted to a control-like profile after successful treatment as was demonstrated from pre- and post-operative samples.

## INTRODUCTION

Colorectal cancer (CRC) is a major global disease burden. In 2012, 693,900 deaths were ascribed to this disease and 1.4 million new cases were estimated to have occurred worldwide [1]. Population-based screenings have successfully reduced CRC related mortality over the past decades in high-income countries, but despite these advances, CRC remains one of the most lethal cancers [1]. In addition, the number of patients diagnosed annually is still increasing due to ageing of the population as well as a small increase in incidence at all ages. Although current screening methods are widely available, there is room for improvement of existing methods and new developments of simple, cost-effective and noninvasive screening tests [2].

Besides early detection, methods for staging with regard to predicting long-term survival are sub-optimal. It is mostly based on the TNM (Tumor-Node-Metastasis) classification, which classifies the anatomic extent of cancer spread and functions as important input for decision making in therapy. However, using this staging system, 25–35% of the patients initially diagnosed with good outcome die within 5 years of diagnosis [3, 4]. Furthermore, in adjuvant setting (stage III) many of the patients receiving chemotherapy do not benefit from the treatment, while experiencing side effects. On the other hand, some patients currently not receiving chemotherapy (stage II) are likely to benefit from the treatment [2, 5]. Moreover, following treatment of CRC, periodic evaluations (monitoring) may lead to the earlier identification and management of recurrent disease. To date, however, no large-scale randomized trials have evaluated postoperative monitoring programs. Therefore, the discovery of new biomarkers that enable earlier detection, prognosis, and/or individualized therapy is essential to improve effectiveness, patient outcomes and financial sustainability of CRC health care.

**Figure 1:**
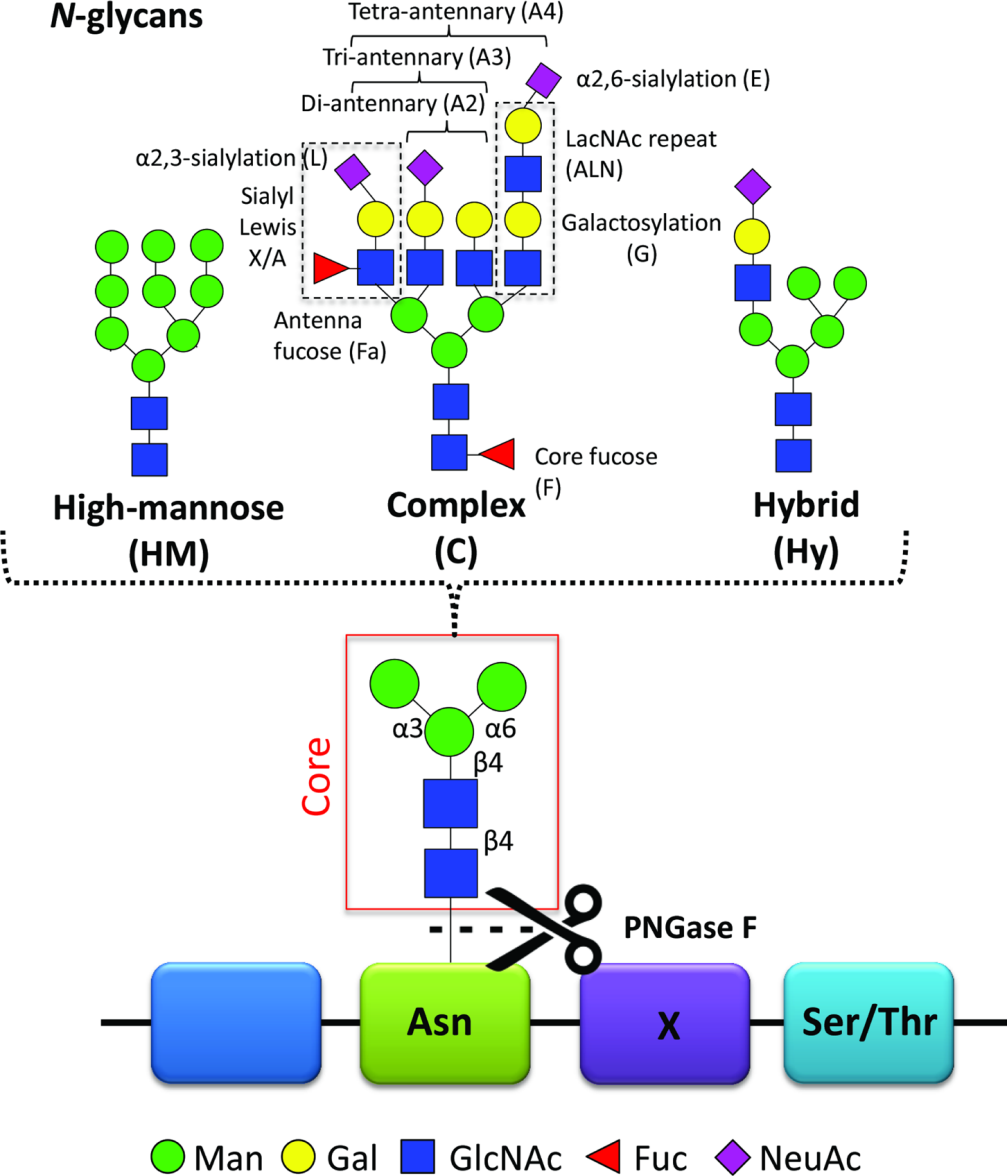
*N-*glycans *N-*Glycans are covalently linked to an asparagine (Asn) of the peptide backbone. The consensus sequence Asn-X-Ser/Thr (Ser = serine, Thr = threonine), where X can be any amino acid except proline, forms a potential *N-*glycosylation site. Importantly, not all potential *N-*glycosylation sites are occupied by *N*-glycans. The enzyme peptide *N-*glycosidase F (PNGase F) can cleave the *N*-glycan off the asparagine. All *N*-glycans consist of a common core-structure of three mannoses (Man) and two *N*-acetylglucosamines (GlcNAc): Man3GlcNAc2. The core can be further elongated by i) only mannoses resulting in high-mannose type *N-*glycans; ii) antennae containing galactose (Gal) and GlcNAc repeats, referred to as LacNAc repeat, antenna- or core-attached fucoses (Fuc) and terminal sialic acids (*N*-acetylneuraminic acid, NeuAc) forming complex type glycans. Depending on the number of antennae added, one distinguishes between di-, tri- and tetra-antennary *N*-glycans; iii) a mixture of the two with one high-mannose arm and one complex-type arm forming the hybrid type *N*-glycan. The illustrated cartoons represent examples, combinations, linkages and the overall extend of the elongations is highly heterogeneous.

Previous studies have reported on cancer-specific changes in protein glycosylation with potential as biomarkers [6–9]. Glycosylation is one of the most common modification on proteins. Proteins that contain one or more covalently linked carbohydrate(s) are referred to as glycoproteins, while the carbohydrates are referred to as glycan(s). Glycoproteins may carry *O*-linked glycans (the carbohydrate is linked via an oxygen atom to the protein) and/or *N*-linked glycans (the carbohydrate is linked via a nitrogen atom to the protein). In the current study, we have focused on the latter type of glycans. These *N*-glycans are attached to the asparagine (Asn) of the peptide backbone in a known consensus sequence, Asn-X-Ser/Thr (X=any amino acid except proline, Ser=serine, Thr=threonine) and are divided in three main types: high-mannose, complex, and hybrid type (Figure 1) [10]. Glycans can alter stability, solubility, size, protease resistance and quaternary structure of the protein to which they are attached and changes in protein glycosylation often affect protein function [10]. This has consequences for many biological processes such as cell-cell interaction, cell-extracellular matrix interaction, cell differentiation and immune response [11]. The biological role of glycans has been comprehensively reviewed [12].

Although the crucial role of glycosylation in pathophysiological processes has been recognized for a long time, at this stage, glycans have not been systematically nor clinically evaluated for diagnostic or prognostic purposes. Levels of carcinoembryonic antigen (CEA) are routinely determined in plasma and serum from CRC patients, but the fact that CEA is a glycoprotein and actually consists of various proteoforms has not yet been taken into account in clinical tests [13]. Currently, only a few glycan antigens are routinely measured as serum biomarker in clinical applications. For CRC, sialyl-Lewis A (CA 19-9; NeuAcα2-3Galβ1-3(Fucα1-4)GlcNAc; NeuAc, *N*-acetylneuraminic acid; Gal, galactose; Fuc, fucose; GlcNAc, N-acetylglucosamine) and its positional isomer sialyl-Lewis X are most frequently evaluated in the clinical setting [13]. Although their role in cancer still needs further elucidation, these glycan epitopes were shown to be elevated in CRC with association for poor prognosis [14, 15]. Other examples of glycan cancer biomarkers are sialyl Tn antigen for gastric cancer and a panel of *N*-glycans that distinguishes ovarian cancer from benign ovarian diseases [16–18]. Various glycan-based serum markers for cancer diagnosis and prognosis are being studied and have been reviewed, including relevant clinical trials [19]. For CRC, however, not many glycan motifs except sialyl-Lewis A and X have been identified in serum.

Deciphering the molecular basis of glycan function and dysfunction has been relatively slow compared to the study of proteins and DNA. This slow progress is due to technical constraints and the fact that glycan biosynthesis is not template-driven like for other biopolymers [20]. Glycans are formed and attached to proteins as a result of the combined activity of glycosyltransferases and glycosidases in the secretory pathway [21]. Changes in the glycosylation affect the appearance of the cell surface as well as secreted proteins. Consequently, glycan-dependent interactions and the resulting cellular processes are influenced, thereby facilitating processes such as the extravasation and invasion of tumor cells, cellular adhesion, tumor progression, and metastasis [22, 23]. These important roles of glycans in cancer formation and progression have made them potential therapeutic targets as well as promising cancer biomarkers [20, 24]. Glycosylation alterations in CRC and associated changes in biological functions are extensive and have been reviewed recently [25].

In the current study, we evaluate the changes in total serum *N-*glycome (TSNG) between CRC patients and matched controls as well as the potential of TSNG to function as a biomarker (panel) for CRC for screening and prognostic purposes. Serum represents a rich source of minimally-invasive biomolecular signatures, of which proteins form a major class. Systematic mapping of protein glycosylation, such as pursued in glycoproteomics and glycomics approaches, receives a growing interest since it is now generally acknowledged that the majority of the serum proteins are glycosylated [26]. Recent advances in mass spectrometry (MS) and related sample preparation strategies have enabled high-throughput measurements of serum *N-*glycans in a robust way. A novel sialic acid derivatization technique has further improved the detection of high-mass and highly sialylated glycans [27]. Here, this novel method was used to investigate CRC associated changes in the serum *N-*glycome in a matched case-control study (124 cases vs. 124 controls). The findings were validated in an independent sample cohort and in post-operative samples from cured CRC cases (both 61 cases vs. 61 controls).

## RESULTS

The total serum *N-*glycome (TSNG) was evaluated with regard to classification potential of CRC in a matched case-control study. Differences in glycan levels in the TSNG between cases and controls were first investigated in the discovery set (124 vs. 124) and findings were validated in two validation sets: i) an independent set of pre-operative case samples vs. control samples (61 vs. 61), ii) the same cases of the first validation set vs. post-operative samples of the same individuals that were cured after surgery (61 vs. 61). The second validation set allowed a comparison of samples with active cancer status and cured status from the same individuals. Descriptive information on the sample sets is summarized in Table 1.

**Table 1:** Cohort characteristics

		Discovery Set		Validation Set		
		Cases (*n* = 124)	Controls (*n* = 124)	Cases^*^ (*n* = 61)	Controls (*n* = 61)	Postoperative^*^ (*n* = 61)
**Female sex, *n* (%)**		56 (45.2)	56 (45.2)	28 (45.9)	28 (45.9)	28 (45.9)
**Age in years, mean (SD)**		66.8 (13.4)	63.0 (10.6)	66.6 (13.3)	63.1 (10.7)	66.6 (13.3)
**Stage, *n* (%)**						
***in situ***		3 (2.4)	n/a	2 (3.3)	n/a	2 (3.3)
**I**		21 (16.9)	n/a	13 (21.3)	n/a	13 (21.3)
**II**		36 (29.0)	n/a	32 (52.5)	n/a	32 (52.5)
**III**		39 (31.5)	n/a	13 (21.3)	n/a	13 (21.3)
**IV**		25 (20.2)	n/a	1 (1.6)	n/a	1 (1.6)

^*^same patients pre- vs. post-operative; n, number of individuals; SD, standard deviation; *in situ*, carcinoma *in situ* (stage 0); n/a, not applicable.

**Figure 2:**
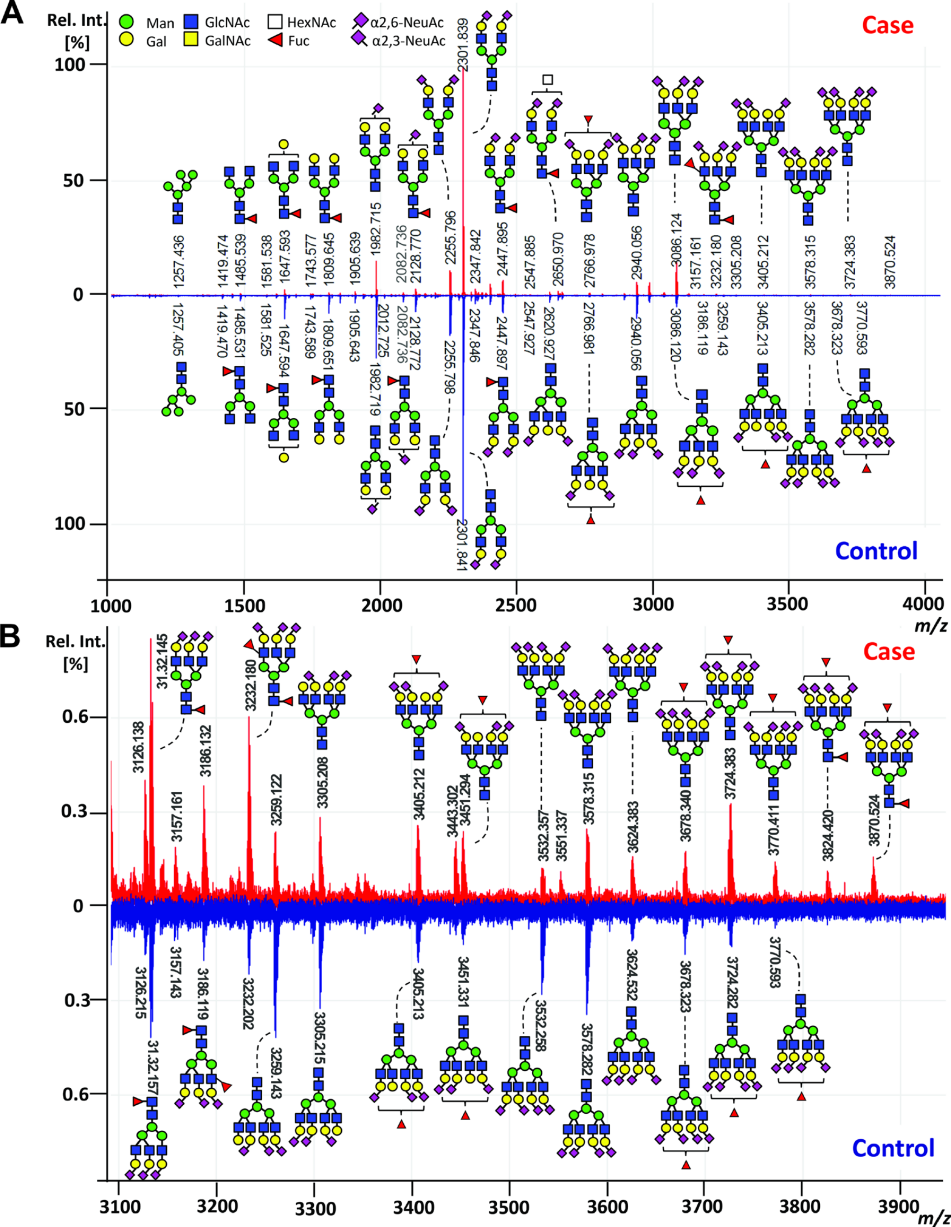
Exemplary MALDI-TOF-MS spectra of the total serum *N*-glycome comparing case and control (**A**) MALDI-TOF-MS spectrum of a case (red) and its matched control (blue) with (**B**) zoom in the higher *m/z* range (*m/z* 3100 to *m/z* 3900). Spectra were recorded in positive ion reflectron mode on a Bruker UltrafleXtreme mass spectrometer. Main peaks were annotated with glycan cartoons reflecting compositions, thereby the presence of structural isomers cannot be excluded. Green circle = mannose, Man; yellow circle = galactose, Gal; blue square = N-acetylglucosamine, GlcNAc; white square = N-acetylhexosamine, HexNAc; red triangle = fucose, Fuc; purple diamond = sialic acid, N-acetylneuraminic acid, NeuAc. Differences in N-acetylneuraminic acid linkages are indicated using angles.

For the glycan analysis, all serum samples were subjected to an enzymatic *N-*glycan release, linkage-specific sialic acid derivatization with clean-up and mass spectrometric (MS) analysis. Exemplary, annotated mass spectra of the serum *N-*glycome of both a case and its matched control are shown in Figure 2. Glycan compositions and structural features were assigned on the basis of MS fragmentation data (Supplementary Figure 1), combined with previously reported assignments [27].

### Data quality

Data on individual *N-*glycans obtained from the discovery set were explored for potential batch effects and confounders such as sample plate, gender, age, and the year of sampling using a cross validated principle component analysis (PCA) with unit variance scaling. Evaluating the distribution of the samples over the plates during the sample preparation showed a batch-effect (Supplementary Figure 2A) which was, however, compensated by the plate design and no batch-correction was performed. Other confounders such as year of sampling category, age category, and sex showed no or minor biases (Supplementary Figure 2B–2D). Importantly, separation between cases and controls was clearly visible in the first and second principal components of this unsupervised PCA model (Supplementary Figure 2E), indicating differences in *N-*glycosylation between the two groups. The Visucon plasma standards as well as the in-house serum standards each clustered closely in the PCA score plot and the coefficient of variation for the main peaks was 11% and 12%, respectively (data not shown).

### Identification of TSNG glycan changes between CRC patients and controls

In order to reveal discriminative *N-*glycans between CRC cases and controls, a two-class (case vs. control) cross validated partial least square-discriminant analysis (PLS-DA) model was fitted and discriminative *N-*glycans were evaluated by their variable influence on projection (VIP) value as well as univariate data analysis. Importantly, cases and controls showed again a separation in the score plot of the first two principle components (Supplementary Figure 2F).

Of the 83 *N-*glycans included in the analysis, 28 were considered as discriminators with PLS-DA VIP-values >1 and *p*-values < 0.01 (Supplementary Table 3A–3D). Of these, 20 could be confirmed in both the case-control and the pre- versus post-operative validation sets (Table 2). Of the discriminating glycans, nine glycans were consistently down-regulated in CRC. Interestingly, all *N-*glycans with decreased expression in CRC compared to controls were of di-antennary nature (Table 2; Supplementary Table 3). Furthermore, many down-regulated discriminators were mono-fucosylated and fragmentation analyses suggests core-fucosylation. In contrast, all eleven *N-*glycans which were up-regulated in CRC were larger structures with three or four antennae and were mostly sialylated (Table 2; Supplementary Table 3).

**Table 2:** Validated colorectal cancer associated serum *N-*glycan alterations

N-Glycans				Discovery set				Validation sets			
		Cases vs. controls				Cases vs. controls		Cases vs. post-operative cases			
Composition	Structure^*^	*m/z* [M+Na]+	Alteration in CRC	Fold Change Case vs Control	VIP	*p*-valuea	Z-score^b^	*p*-value^a^	Z-score	*p*-valuec	Z-score
**H4N4F1**	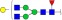	1647.59	down	**0.73**	1.5	4.00E−08	−5.5	1.00E−03	−3.3	1.00E−03	−3.2
**H5N4**	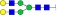	1663.58	down	**0.72**	1.6	2.80E−08	−5.6	4.00E−04	−3.5	7.00E−03	−2.7
**H5N4F1**	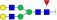	1809.64	down	**0.59**	2.0	3.50E−15	−7.9	2.20E−05	−4.2	4.00E−03	−2.9
**H4N4E1**	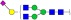	1820.66	down	**0.81**	1.4	2.80E−06	−4.7	4.00E−03	−2.9	5.70E−02	−1.9
**H4N5F1**	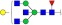	1850.67	down	**0.81**	1.2	4.40E−07	−5.1	1.20E−05	−4.4	1.50E−04	−3.8
**H5N5F1**	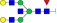	2012.72	down	**0.80**	1.3	1.50E−07	−5.3	4.80E−04	−3.5	2.00E−04	−3.7
**H5N4F1L1**	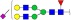	2082.72	down	**0.81**	2.1	1.60E−09	−6	5.00E−05	−4.1	1.10E−05	−4.4
**H5N4F1E1**	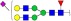	2128.77	down	**0.77**	1.9	9.30E−13	−7.1	1.00E−03	−3.2	1.00E−03	−3.4
**H5N4F1L2**	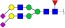	2355.81	down	**0.89**	1.6	2.40E−04	−3.7	3.00E−03	−2.9	7.80E−02	−1.8
**H6N5F1E1L1**	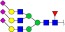	2766.98	up	**1.23**	1.1	2.90E−04	−3.6	2.00E−04	−3.7	4.50E−06	−4.6
**H6N5E3**	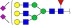	2986.09	up	**1.27**	1.4	2.90E−06	−4.7	2.00E−03	−3.1	1.00E−04	−3.9
**H6N5F1E1L2**	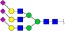	3040.07	up	**1.31**	1.3	1.70E−05	−4.3	7.30E−05	−4	8.90E−06	−4.4
**H6N5F1E2L1**	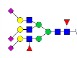	3086.11	up	**1.48**	1.4	4.40E−07	−5	7.00E−05	−4	2.50E−07	−5.2
**H6N5F1E3**	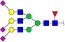	3132.15	up	**1.37**	1.5	1.90E−06	−4.8	2.00E−04	−3.7	4.90E−05	−4.1
**H6N5F2E1L2**	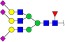	3186.13	up	**1.39**	1.2	1.30E−04	−3.8	2.20E−04	−3.7	2.20E−06	−4.7
**H6N5F2E2L1**	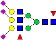	3232.17	up	**1.38**	1.3	2.10E−05	−4.3	1.20E−04	−3.8	4.7E−05	−4.1
**H7N6F1E1L2**	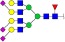	3405.20	up	**1.29**	1.2	5.00E−05	−4.1	3.00E−05	−4.2	8.70E−05	−3.9
**H9N8E1**	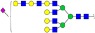	3443.24	up	**1.27**	1.2	1.00E−03	−3.4	3.50E−04	−3.6	3.10E−05	−4.2
**H7N6F1E2L1**	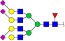	3451.24	up	**1.29**	1.4	1.00E−03	−3.4	7.50E−05	−4	1.40E−04	−3.8
**H7N6F1E2L2**	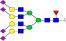	3724.33	up	**1.43**	1.4	3.20E−06	−4.7	1.30E−05	−4.4	3.60E−05	−4.1

^*^structural isomers cannot be excluded

^a^Mann-Whitney *U* test, *p*-value < 0.01

^b^Z-score: indicates how many standard deviations an element is from the mean

^c^Wilcoxon signed ranks test, *p*-value < 0.1; *m/z,* mass to charge ratio; M, mass; Na, sodium; VIP, variable importance in projection; CRC, colorectal cancer; H, hexose; N, *N*-acetylhexosamine; F, fucose; E, α2,6-linked sialic acid; L, α2,3-linked sialic acid.

**Figure 3:**
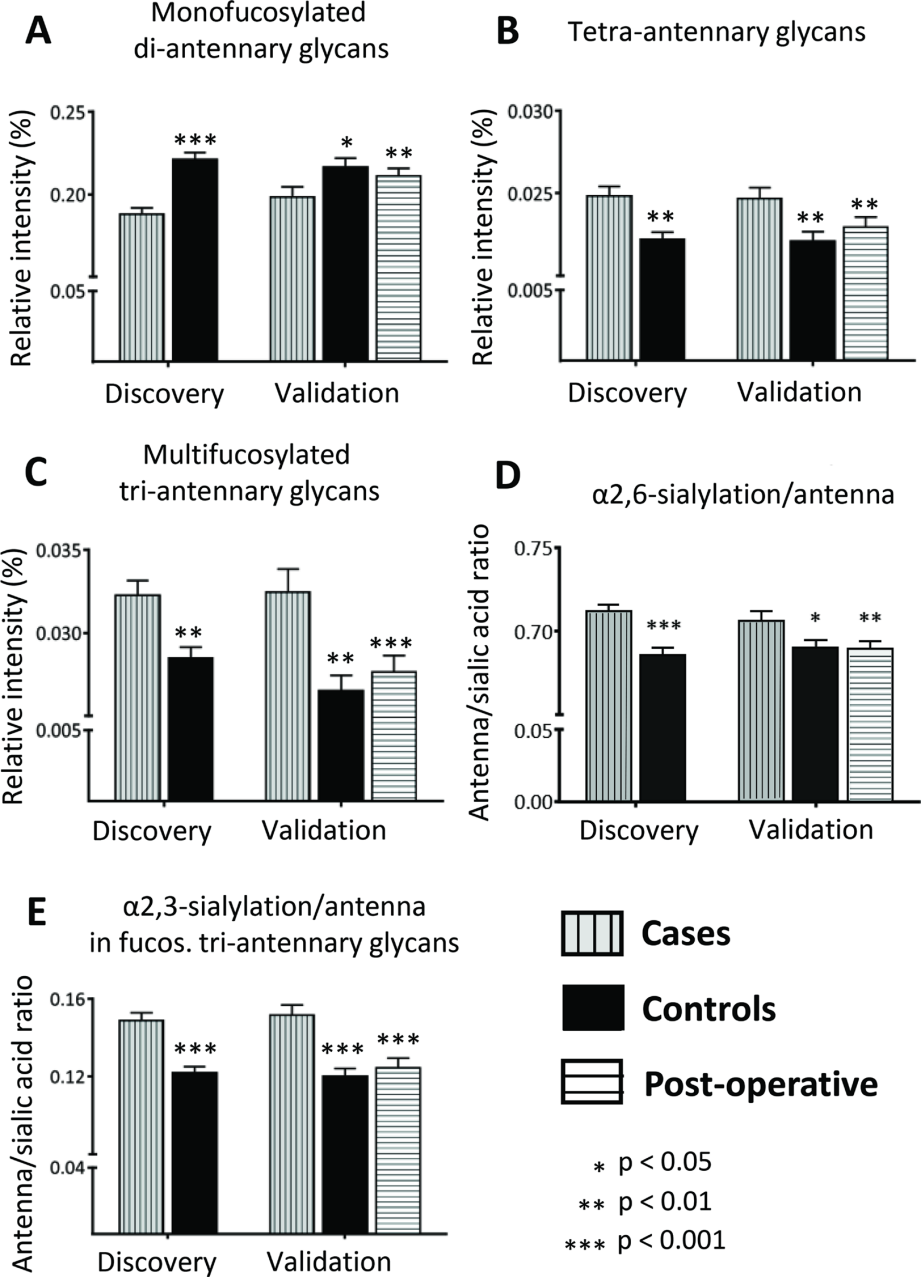
Alterations in derived *N*-glycan traits Derived *N*-glycan traits were calculated to evaluate glycosylation changes between CRC and healthy controls. Significant changes were observed for (**A**) Mono-fucosylated di-antennary glycans, which were lower in CRC as compared to controls, (**B**) Tetra-antennary glycans, (**C**) Multi-fucosylated tri-antennary glycans, (**D**) α2,6-sialylation/antenna and (**E**) α2,3-sialylation/antenna in fucosylated tri-antennary glycans, which were all elevated in CRC. Differences were evaluated by Mann-Whitney *U* test for the discovery and the first validation set. The third evaluation consisted of paired univariate analyses in the postoperative validation set using the Wilcoxon Signed Ranks test. In the latter comparison, preoperative sera from the cases in the validation set were compared to postoperative sera from the same individuals, but now with a cancer free status. Univariate analyses were performed in SPSS (Version 20, IBM, NY, USA). Boxplots consistently indicate the mean ± standard error (SE). *Significances are indicated and correspond to* ^*^ *p-value < 0.05,* ^**^ *p-value < 0.01,* ^***^ *p-value < 0.001.*

To investigate whether the discriminators reflect only single glycan changes or overall changes in structural features, derived traits were calculated and analyzed which sum the *N-*glycans sharing particular features such as galactosylation, sialylation, fucosylation. Significant differences were found for the derived trait of mono-fucosylated di-antennary glycans which were decreased in CRC (fold change 0.85, *Z* = −5.4, *p*-value = 6.1E-08 for discovery set; Figure 3A, Supplementary Table 4), reflecting observed differences from individual *N-*glycan changes described above. Discriminating *N*-glycans which were elevated in the CRC patient serum were characterized by higher numbers of antennae. In line, tetra-antennary *N*-glycans in CRC (fold change 1.12, Z = −3.1, *p*-value = 0.002 in discovery set; Figure 3B, Supplementary Table 4) as well as multi-fucosylated tri-antennary glycans were increased in CRC as compared to controls (fold change 1.12, Z = −2.9, *p*-value = 0.004 for discovery set; Figure 3C, Supplementary Table 4). Furthermore, all up-regulated glycan discriminators were characterized by at least one α2,6-linked sialic acid and, accordingly, α2,6-sialylation was significantly elevated in CRC in all corresponding traits, independent of the antennarity or fucosylation status (Figure 3D, Supplementary Table 4). In contrast, α2,3-linked sialylation was specifically increased in fucosylated tri-antennary glycans for the CRC samples (fold change 1.22, Z = −4.7, *p*-value = 2.1E-06 for discovery set; Figure 3E, Supplementary Table 4), indicative of an increase in sialyl Lewis epitopes on tri-antennary glycans. Interestingly, the galactosylation per antenna was mostly elevated for tri-antennary glycans with fucosylation, which suggests that the observed increase in sialylation for this group may be due to an increase in substrate (galactose) for the corresponding sialyltransferases (Supplementary Table 4).

The comparison between individual *N*-glycan discriminators and derived traits indicates that glycan alterations are shared by a panel of structurally related glycans. Therefore, the potential of the TSNG as panel of potential markers was further evaluated instead of focusing on individual glycans or derived traits.

### Case-control classification based on serum N-glycome analysis

**Figure 4:**
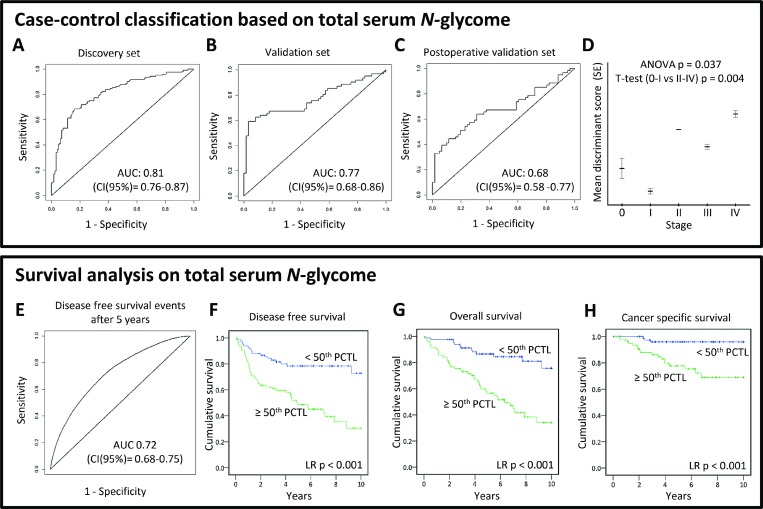
(TOP PANEL) Case-control classification based on total serum *N-*glycome analysis To evaluate the diagnostic potential of total serum *N-*glycome analysis in CRC, logistic ridge regression was used to calibrate a discriminant rule in the discovery dataset. The discriminative performance of this score was evaluated internally after double cross-validation in the discovery set (**A**), and externally in both validation (**B**, **C**) sets by ROC curves. Furthermore, the association of the total serum *N-*glycome-derived discriminant score and its association with disease stage (**D**) was evaluated. *AUC, area under the curve; CI, 95% confidence interval;* (BOTTOM PANEL) Survival analyses based on total serum *N*-glycome. A prognostic score based on the total serum *N-*glycome as a panel of markers was calculated in the curatively treated cohort using cox regression with ridge penalization. Age and stage were included in the model as fixed effects. The performance of the resulting double cross-validated prognostic score was evaluated by time-dependent ROC curves, constructed for follow-up time points 1, 5 and 10 (**E**) years. Kaplan-Meier plots are shown estimating the (**F**) disease free survival, (**G**) overall survival, and (**H**) cancer specific survival for two groups created by dichotomizing the double-cross validated prognostic score on the median. *AUC, area under the curve; CI, 95% confidence interval; PCTL, percentile.*

To evaluate case-control classification based on the TSNG, a logistic ridge regression was used to calibrate a discriminant rule in the discovery dataset. The obtained discriminant score derived from the TSNG allowed the differentiation between cases and controls and its discriminative performance in the discovery set after double cross validation was good with an AUC of 0.81 (95% confidence interval (CI): 0.76–0.87; Figure 4A). The corresponding specificity and sensitivity at the optimal case probability score cut-off in the discovery set were 0.79 and 0.72, respectively. The discriminant rule was then applied on the validation set (external validation) and showed moderate performance with AUC 0.77 (95% CI: 0.68–0.86; Figure 4B) and a specificity and sensitivity of 0.82 and 0.67, respectively. Last, also the intra-individual analysis of pre- and post-operative samples of cured cases could be discriminated using the TSNG score, but the performance decreased further (AUC 0.68; 95% CI: 0.58–0.77; specificity 0.69, sensitivity 0.64; Figure 4C). Importantly, the discriminant scores were significantly associated with disease stage with lower scores in early CRC stages (Figure 4D), suggesting a sequential change of the *N-*glycome during cancer progression which could aid decisions on patient stratification in combination with the current staging system. Although the performance of the TSNG-based glycan analysis was promising with regard to classification, these results do not render early diagnosis feasible since the number of early stage cancer patients in this cohort is rather limited (see Table 1). Therefore, we have primarily focused on the discovery of *N*-glycan changes between CRC cases and controls and their value as prognostic marker.

### Observed glycan alterations predict stage- and age-independent survival

Survival analyses were performed on all cases of the cohort to investigate which of the above described glycan alterations were associated with prognosis/survival in this CRC cohort. Of the 20 serum *N-*glycans aberrantly expressed in CRC patient serum, 12 were associated with overall survival (OS; *p*-value < 0.05; Supplementary Figure 3A–3L; Supplementary Table 5). Of the glycans with prognostic potential, one was down-regulated and eleven were up-regulated in CRC. Increased serum levels (>70th percentile) of the in CRC downregulated glycan (Hex4HexNAc4(α2,6)NeuAc1; Supplementary Figure 3A) were associated with longer survival, while the elevation of the other glycans associated with shorter survival. All glycans remained significantly associated with survival after adjusting for age and eight remained significant when adjusted for both age and stage (Supplementary Table 7). Furthermore, nine of the 12 glycans which associated with overall survival were also significantly associated with cancer-specific survival (CSS; Supplementary Figure 4A–4F).

In order to test the potential of the TSNG as prognostic marker, cox regression with ridge penalization was used for the calibration of a prognostic score based on relative glycan intensities of the TSNG on the curatively treated cohort. Solely the curatively treated patients were used for the calibration to show potential relevance for adjuvant treatment decision making. Age and stage were also included in the model as fixed effects and the linear predictor is proposed as prognostic score, which showed good performance with an AUC for discriminating disease-free survival (DFS) events from censoring after 5 years follow-up of 0.72 (95% CI: 0.68–0.75; Figure 4E). Kaplan–Meier plots for the on the median dichotomized prognostic score are shown in Figure 4F–4H. At 5 years of follow-up, only 46% of patients with a high (≥50th percentile) TSNG prognostic score were alive, against 87% for the curatively treated cases with a low TSNG prognostic score. Confidence intervals (CI) for DFS AUCs built with a prognostic score including age, stage and TSNG (CI AUC at 5 years: 0.68–0.75) and a prognostic score solely derived from age and stage (CI AUC at 5 years: 0.61–0.66) did not overlap. This indicates that the TSNG significantly added to the prognostic value beyond age and stage.

## DISCUSSION

With our novel approach, we have identified several serum *N-*glycans that have the potential to function as a biomarker for CRC. Discriminative *N*-glycans elevated in CRC as compared to controls were mainly characterized by higher branching, resulting in tri- and tetra-antennary *N*-glycans, as well as higher sialylation. This is in line with previous findings associating the increase of β1,6-branching as well as sialylation with invasion and metastasis in CRC. *N*-glycans with increased β1,6-branching were further described to be involved in regulation of cell proliferation and differentiation and were suggested as markers to predict aggressiveness of CRC tumors based on tissue and cell line studies, but little is known about their role in serum [28]. A recent study by Doherty *et al.* investigating serum *N*-glycan changes in CRC patients compared to controls confirmed the increased expression of larger, mostly sialylated *N*-glycans (tri- and tetra-antennary) using UPLC hydrophillic interaction liquid chromatography (HILIC) with fluorescence detection [29]. In contrast, *N*-glycans with reduced expression in CRC were di-antennary and mainly core-fucosylated. Strikingly, four out of seven down-regulated discriminators were also found to be down-regulated in CRC patient tissues as compared to control tissues from the same individuals [30]. In line, the study by Doherty and co-workers confirmed the decreased expression of core fucosylated di-antennary in serum from CRC patients [29]. Furthermore, *N*-glycans with decreased expression in CRC were main *N*-glycans found on IgG for which decreased expression in CRC as well as inflammatory bowel disease have been described [31, 32]. In line with these results, Zhao *et al.* found core-fucosylated di-antennary *N*-glycans to be decreased in serum from CRC patients and patients with colorectal adenoma when compared with the serum *N*-glycome of healthy controls [33]. Their results showed the potential of *N*-glycan marker-based diagnostic models for CRC, which outperformed CEA. Their approach, however, used DNA sequencer-assisted/fluorophore-assisted carbohydrate electrophoresis (DSA-FACE) which required the removal of sialic acids prior to the analysis and allowed the detection of a limited number of *N*-glycans (*n* = 9). In contrast, the here applied state-of-the-art MS-based glycomics approach allows for high-throughput analysis and was shown to be robust with good repeatability [27]. It further includes a linkage-specific stabilization of sialic acids that increases the effective depth of analysis (broader coverage of different glycan structures) and adds important glycobiological information in the analysis of large sample cohorts. In order to translate such glycan biomarkers into the clinic, the methods need to be straight-forward and easy-to-use. Therefore, we are working on the automation of the glycomics sample preparation [34, 35], thereby aiding sample handling and robustness, though high-end instrumentation is still needed. Transferring the method to other systems is as well a possibility and to this end, routinely applied triple quadrupole mass spectrometers or MALDI-platforms that are already in use for bacteriotyping are a good alternative.

With our TSNG analysis, cases could be successfully discriminated from controls and furthermore survival in CRC could be predicted. Various *N*-glycans were found which were altered in CRC and had prognostic potential. These glycans may contribute to individualized therapy decisions as most prognostic glycans remained significant after adjustment for age and stage. To increase the robustness of our method as well as to investigate if the TSNG as a panel of biomarkers can aid in specificity, the prognostic potential of the TSNG was evaluated in patient samples after curation. The prognostic rule, calibrated on the TSNG of curatively treated cases, showed a difference in stage- and age-independent survival. The TSNG derived prognostic information detected in pre-operative serum opens new insights for operative and neoadjuvant treatment strategies. The technique used in this study easily facilitates the use of a panel of glycans that can be measured simultaneously–in this case the TSNG–which can increase robustness of the method. It further facilitates interpretation and analysis of glycomics data since once the panel of glycans is established and peaks in the mass spectra annotated, the information can be transferred to other samples, supporting a transfer to a clinical routine. It should be noted that prognostic biomarkers are not necessarily cancer-specific. Therefore, the here observed glycan alterations could also offer prognostic potential in other cancers which should be explored in a pan-cancer study.

While the TSNG scoring showed potential as prognostic marker for CRC, the early detection performance was limited and not scope of this study. While total cases and controls could be discriminated, and showed significant differences, no evident changes were seen in stage I and stage 0 (carcinoma *in situ*), resulting in a moderate discriminative capability. The superior performance of the discriminant rule in the discovery set might thus be caused by the higher proportion of cases with advanced CRC stages as compared to the pre-/post-operative comparison of cured cases which were mainly in earlier stages. The differences in clinical characteristics of the cases between the discovery and validation set were inevitable due to the study design and probably resulted in an underestimation of the accuracy of the discriminant rule in de validation set. Importantly, the validation set is a better reflection of a screening population, with few late stage disease cases. Most late stage patients are symptomatic and will be enrolled to the health system through another route than screening. Therefore, further studies, dedicated for early detection, are needed to evaluate the potential of TSNG in this respect, taking into account that the study cohort should reflect the target population with mainly stage 1 and stage 2 cases. Ideally, early-diagnosis studies have a longitudinal study design in which individual changes can often be picked up at an earlier stage than in retrospective cohorts that contain both early and late stage cases. The potential of longitudinal studies has been recently shown a proof of concept towards personalized medicine [36] and might be especially suited for monitoring patients with hereditary cancer.

Furthermore, with our method, *N-*glycans released from ‘all’ serum glycoproteins were analyzed together. Though the strategy of building scores based on the TSNG showed great potential, the addition of an extra fractionation step by selecting specific glycoproteins or glycoprotein families might further improve clinical accuracy. Choosing potential glycoprotein candidates, however, remains difficult since the carriers of the glycans which were altered in CRC are not exactly known and may often be a mixture of numerous different glycoproteins. Glycopeptide-centered glycoproteomics, in which glycans remain attached to protein fragments for analysis, might provide useful information to identify carrier proteins, but also presents a technical challenge for such a complex mixture.

Antibodies (IgG, IgA and IgM), transferrin and alpha-2-macroglobulin represent together approximately 75% of all serum glycoproteins. The remaining part mainly consists of alpha-1-antitrypsin, alpha-1-acid glycoprotein, haptoglobin, ceruloplasmin, the complement system, and apolipoproteins–making the liver and plasma B-cells the main source of serum glycoproteins [37]. Based on the observed changes in combination with our recent review mapping *N*-glycan contributions to serum glycoproteins, alpha-1-acid glycoprotein and IgG might be promising candidates [38]. More information on the origin of the glycan alterations could not only lead to improved accuracy for early CRC detection, prognostic capabilities and prediction of therapeutic response, but also to understand cancer associated glycan changes in serum.

During inflammation, the production of acute phase proteins is increased which leads to their higher abundance in the serum, but also cytokines produced by the tumor microenvironment can stimulate the hepatocytes in the liver [39]. In our study, we observed an increase in tetra-antennary *N*-glycans as well as α2,3-sialylation of fucosylated tri-antennary glycans, indicating the presence of sialyl Lewis epitopes. Increased expression of sialyl Lewis antigens especially on tri- and tetra-antennary glycans has been reported on haptoglobin, alpha-1-acid glycoprotein and other acute phase proteins for inflammatory conditions as well as cancer [38, 39]. This raises the question of the relation between inflammation and cancer, which was comprehensively addressed in a recent review [38]. In that review, it was furthermore hypothesized that the increase of tri- and tetra-antennary *N*-glycans, together with increased expression of sialyl Lewis antigens represented a systemic side effect of cancer-promoted inflammatory cytokines [38].

In conclusion, the here presented clinical glycomics study shows that *N*-glycan changes observed in the serum of CRC patients are powerful novel biomarkers for prognosis which could aid in discriminating patients which are likely to be disease-free and patients likely experiencing recurrence, thereby promoting individualized treatment decisions in colorectal cancer. Further, we could show the potential of using a scoring system based on TSNG serving as biomarker panel which can increase the specificity and overcome the limitation of single protein markers currently used in the clinic. It is noted that translation of such a glycan biomarker panel should be accompanied by additional method development and instrument simplification/automation.

## MATERIALS AND METHODS

### Clinical study design

Serum samples were collected by the Leiden University Medical Center (LUMC) Surgical Oncology Biobank between October 2002 and March 2013 according to a standardized protocol. This study was approved by the Medical Ethics Committee of the LUMC and was performed in accordance to the Code of Conduct of the Federation of Medical Scientific Societies in the Netherlands (http://www.federa.org/). Serum samples from 185 CRC patients sampled prior to any treatment were selected as cases and serum from 185 volunteers, mainly the partners accompanying the patients and therefore closely reflecting the general population, were selected as controls. Controls were matched to cases for sex, year of sampling, and age. The study samples were divided into a discovery set (124 cases vs 124 controls) and an independent validation set (61 cases vs 61 controls). Cases were only selected for the validation set if a ‘cancer free’ post-operative sample was available. These post-operative samples enabled the comparison of *N-*glycan profiles between cancer positive and cancer-free serum originating from the same individual. A cancer-free post-operative sample was defined as serum sampled after a post-operative wash out period of 45 days, from a curatively treated case without recurrence and with adequate follow-up (>1393 days). The cut-off for this follow-up period was chosen based on the longest time interval between surgery and oncologic event (cancer recurrence or cancer related death) in the study. Disease free survival (DFS) was defined as time to any event, irrespective of cause. Time to death caused by the same cancer was defined as cancer specific survival (CSS). Besides death due to the original tumor, this included deaths due to a second primary tumor of the same cancer. Overall survival (OS) was calculated as time to death, irrespective of the cause [40]. All clinical information underwent a reversible anonymization before it was merged with a biobank database. Apart from sample characteristics, only the date of birth and sex was recorded from the presumed healthy volunteers that served as controls. Both pre-operative as post-operative serum samples from cases had been stored at 20° C after centrifugation until monthly biobank collection and storage at 80° C, while serum samples from controls were stored directly at −80° C after centrifugation. Previously, it has been reported that glycans are not susceptible to changes in tubes used for sample storage or processing methodology [41]. Our long-term experience with glycan sample storage corroborates with these findings and has not pointed towards any type of glycan degradation during biobanking. More information on the clinical study design is described in the Supplementary Information and cohort characteristics are summarized in Table 1. Blanks (water) and two different in-house standards (i) pooled serum from four volunteers and (ii) control Visucon-F plasma pool (citrated and 0.02 M HEPES buffered plasma pool from 20 healthy human donors; Affinity Biologicals, Ancaster, Canada) were used for quality control. More details on sample collection and storage are described in Supplementary Information.

### Experimental study design

All 431 study serum samples (2 × 124 + 2 × 61 + 61), 28 blank samples and 46 in-house standard serum samples were distributed over seven 96-well plates using a randomized block plate design. The randomization process resulted in an equal distribution with regard to the variables age, stage, sex and year of sampling over the plates. Furthermore, the design ensured that cases were on the same plate as their paired controls and corresponding post-operative samples. *N-*glycans were enzymatically released from all serum glycoproteins using peptide *N*-glycosidase F (PNGase F). Subsequently, glycans were derivatized using the ethyl esterification protocol as described previously [27], resulting in the stabilization of sialylated glycans in a linkage-specific manner. With this protocol, sialic acids attached to the third carbon atom of galactose (α2,3-linked sialic acids, L) will form an intramolecular lactone, while sialic acids attached to the sixth carbon atom of galactose (α2,6-linked sialic acids, E) are esterified, leading to characteristic mass shifts enabling their discrimination. After derivatization, glycans were purified and enriched using hydrophilic interaction liquid chromatography solid phase extraction (HILIC-SPE) using Sepharose beads in 96-well format. Matrix assisted laser desorption ionization time-of-flight (MALDI-TOF) MS was used for identification and relative quantification of glycans. Fragmentation analyses to confirm glycan compositions and structural features were performed on all peaks. A detailed description of the materials and experimental as well as data processing procedures is provided in the Supplementary Information (Supplementary Tables 1, 2 and 6).

### Derived glycan traits

Individual glycans can be grouped based on their structural characteristics. The resulting glycan groups, so-called derived glycan traits, facilitate interpretation since they summarize the total serum *N-*glycome (TSNG) and reflect certain aspects of glycobiology. The first set of derived traits partitioned all glycans into high-mannose-, complex- and hybrid-type glycans. Complex-type glycans were further subdivided and analyzed according to the number of antennae, fucosylation, galactosylation and sialylation. Antenna and fucosylation traits were calculated by summing the relative intensities of the selected glycans [35, 42]. Galactosylation and sialylation were expressed per antenna. All derived glycan traits were calculated in SPSS (Version 20, IBM, NY) as outlined in Supplementary Table 1A–1G.

### Data analysis

Cross validated principal component analysis (PCA) and cross validated partial least squares discriminant analysis (PLS-DA) were performed in SIMCA software V13 (Umetrics AB, Umea, Sweden). Univariate analyses and survival analyses of individual glycans were performed in SPSS (Version 20, IBM). Single glycan alterations, *i.e.* unimarker intensity changes, in the discovery dataset were considered statistically significant and validated if the PLS-DA variable importance in the projection (VIP) value was > 1 in the discovery data and if the *p*-value was < 0.01 in both the discovery and validation set [43]. Significant and validated unimarkers were furthermore evaluated for their prognostic capacity. A double cross validated ‘discriminant’ rule based on all glycan intensities, *i.e.* a biomarker signature approach, was calibrated in the discovery dataset using logistic ridge regression in order to evaluate the diagnostic potential of TSNG-based classification. Discriminative performance was evaluated in the discovery set and in both validation sets by ROC curves. Cox regression with ridge penalization was used for the calibration of a double cross validated ‘prognostic’ rule based on all relative glycan intensities of the curatively treated cohort. Age and stage were included in the model as fixed effects and the linear predictor is proposed as prognostic score. Performance of the resulting age- and stage-independent prognostic score was evaluated by time-dependent ROC curves. A detailed description of the data analysis is given in the Supplementary Information.

## SUPPLEMENTARY MATERIALS FIGURES AND TABLES













## Abbreviations

CI: confidence interval
CRC: colorectal cancer
CSS: cancer specific survival
DFS: Disease free survival
E: α2,6-linked *N*-acetylneuraminic acid
Fuc: F: fucose
Gal: galactose
GlcNAc: *N*-acetylglucosamine
Hex: H: hexose
HexNAc: N: *N-*acteylhexosamine
HILIC-SPE: hydrophilic interaction liquid chromatography solid phase extraction
L: α2,3-linked *N*-acetylneuraminic acid
MALDI-TOF: Matrix Assisted Laser Desorption/Ionization time-of-flight
MS: mass spectrometry
NeuAc: S: *N*-acetylneuraminic acid
OS: Overall survival
PCA: principle component analysis
PLS-DA: partial least squares discriminant analysis
PNGase F: peptide *N*-glycosidase F
TSNG: total serum *N*-glycome
