# Supplementary material for: Serum N-glycome alterations in colorectal cancer associate with survival
Source: Oncotarget. 2018 Jul 17;9(55):30610–23. doi: 10.18632/oncotarget.25753 (PMC6078140; doi:10.18632/oncotarget.25753)
Supplement: Supplementary file 1 [file oncotarget-09-30610-s001.pdf]

Serum *N*-glycome alterations in colorectal cancer associate with survival

SUPPLEMENTARY MATERIALS

Experimental design

Chemicals and solvents

Sodium dodecyl sulfate (SDS), disodium hydrogen phosphate dihydrate ($Na_2HPO_4 \times 2H_2O$) potassium dihydrogen phosphate (KH_2PO_4) and sodium chloride (NaCl) were purchased from Merck (Darmstadt, Germany). Nonidet P-40 substitute (NP-40), 1-hydroxybenzotriazole (HOBt) and trifluoroacetic acid (TFA) were bought from Sigma-Aldrich (Steinheim, Germany). Super-DHB (SDHB), a 9:1 mixture of 2,5-dihydroxybenzoic acid and 2-hydroxy-5-methoxybenzoic acid, was also obtained from Sigma-Aldrich (Steinheim, Germany). One-ethyl-3-(3-(dimethylamino)propyl)- carbodiimide (EDC) came from Fluorochem (Hadfield, United Kingdom). Acetonitrile high performance liquid chromatography supra-gradient (ACN) was acquired from Biosolve (Valkenswaard, The Netherlands). Peptide *N*-glycosidase F (PNGase F) was purchased from Roche Diagnostics (Mannheim, Germany). Calibration peptide mix for MALDI-TOF-MS measurements was obtained from Bruker Daltonics (Bremen, Germany). All water (MQ) used in this study was purified (resistivity ≥ 18.2 MΩ·cm at 25° C) using the PURELAB Ultra Genetic water purification system (ELGA LabWater).

Sample collection and storage

All samples were collected by the LUMC Surgical Oncology Biobank between October 2002 and March 2013 according to a standardized protocol. All samples were drawn in serum tubes (8.5 mL BD Vacutainer SST II plastic serum tubes, BD, Plymouth, United Kingdom) and centrifuged within maximally 4 hours from blood sampling. The samples were stored frozen until aliquoting. Cases, both preoperative and postoperative serum samples, have been frozen at −20° for a maximum of 30 days before biobank collection and storage at −80° C. Controls have been stored directly at −80° C. On a regular basis, all samples collected in the preceding period were aliquoted in 8-fold conform a strict and automated procedure. Samples were thawed on ice and aliquoted automatically using an 8-channel Microlab STAR line pipetting robot (Hamilton, Bonaduz, Switzerland) in sterile barcoded tubes (500 µl, 2D Barcoded, V Bottom Tubes, Thermo Scientific, Hudson). These aliquots were stored in barcoded racks at −80° C until further sample processing.

Glycan release

N-glycans were released from the serum proteins similar to a previously described method for human plasma samples [1]. Two microliters of serum were denatured by adding 4 µL of 2% SDS, followed by centrifuging for 1 min at 200 RCF, shaking for 5 min on a horizontal plate shaker at 1000 rpm (Titramax 100, Heidolph Instruments, Schwabach, Germany), and incubation for 10 min at 60° C. Subsequently, glycans were released by adding 4 µL of release mixture containing 2% NP-40 and 0.2 µL PNGase F in 2.5× phosphate buffered saline (14.25 g/L $Na_2HPO_4 \times 2H_2O$, 1,25 g/L KH_2PO_4 and 21,25 g/L NaCl) followed by 5 min on a horizontal plate shaker and incubation overnight at 37° C.

Linkage specific sialic acid derivatization.

Glycans were derivatized by ethyl esterification, causing stabilisation of sialylated species in a linkage specific manner, according to a former published in house developed high throughput method [2]. First, 20 µL of ethylation reagent (250 mM EDC 250 mM HOBt in ethanol) was added to each well of a microtitration plate (96-well PCR plate, polypropylene, Greiner Bio-One, Alphen a/d Rijn, The Netherlands). Subsequently, 1 µL of glycan released sample was added to each well. To prevent evaporation the plate was covered with foil before incubation at 37° C for one hour. This procedure was performed in duplicate for each plate. After incubation, 20 µL of ACN was added to each well.

Sample purification and glycan enrichment

Hydrophilic interaction liquid chromatography solid phase extraction (HILIC-SPE) was performed using agarose beads (Cl-4B Sepharose beads, 45−165 μm, Sigma-Aldrich, Steinheim, Germany), according to a slightly modified version of a previously described method [2, 3]. Thirty microliters of agarose beads were added in 20% ethanol to a 96-well filter plate (0.7 mL/well, PE frit, Orochem, Naperville, Illinois). Solvent was removed using a $\text{MultiScreen}_{\text{HTS}}$ Vacuum Manifold (Millipore, Billerica, Massachusetts). Beads were washed three times with 200 μL MQ and three times with 200 μL 85% ACN on the vacuum manifold. Residual solvent and the released and derivatized glycan samples were loaded onto the plate and shaken for 1 min on a horizontal plate shaker. The remaining solvent was removed by slow vacuum. Next, the plate was washed three times with 200 μL 0,1% TFA/85% ACN and three times with 85% ACN. During the last washing step of each washing solution, the plate was incubated for 15 min on a horizontal plate shaker at 1000 rpm. Glycans were eluted from the beads with 30 μL MQ by incubating for 5 min on a horizontal plate shaker at 1000 rpm, followed by centrifuging for 1 min at 200 RCF.

Mass spectrometry

Two microliter of HILIC SPE purified sample was spotted on a MTP AnchorChip 800/384 T target (Bruker Daltonics). Each sample was spotted in duplicate, which together with the reiterated derivatization step resulted in four mass spectra for each sample. Sample was mixed on the target with 1 μL SDHB (5 mg/mL in 50% ACN/1 mM NaOH) and left to dry by air. The dried spots were recrystallized using 2 μL ethanol, causing centralisation and uniformity of the crystals. Mass spectrometry analyses were performed on an UltrafleXtreme matrix assisted laser desorption ionisation time of flight (MALDI-TOF) mass spectrometer equipped with a Smartbeam-II laser and FlexControl software version 3.4.135.0 (Bruker Daltonics). Peptide calibration standard (Bruker Daltonics) was used for external calibration. For each spectrum, 10 000 laser shots were accumulated using a complete sample random walk with 200 shots per raster spot, at a laser frequency of 2000 Hz. Mass window was set to *m/z* 1000 to 5000 with matrix suppression up to *m/z* 900. Tandem mass spectrometry (MALDI-TOF/TOF-MS/MS) was performed on the most abundant and most discriminating peaks using laser-induced disassociation.

Data processing

All raw mass spectra underwent baseline subtraction and were exported as XY files using the batch process function of FlexAnalysis (version 3.3 build 65, Bruker Daltonics). In FlexAnalysis, all individual profiles of the first plate as well as the sum spectra of all seven plates were internally calibrated using the calibrants listed in Supplementary Table 6. For this purpose, an in-house developed Python script (version 2.7.8, Python Software Foundation; http://docs.python.org/py3k/reference/index.html) was used to create sum spectra for each of the seven plates measured. All peaks (isotope clusters) with a signal to noise ratio (S/N) of three and good isotopic pattern were listed. These peaks (analytes) were annotated with glycan compositions in GlycoWorkbench (version 2.1 stable build 146) using the Glyco-Peakfinder tool [4]. The exported baseline subtracted XY files were further processed using the composition list and in-house developed software (MassyTools, version 0.1.6.4) [5]. Internal calibration of the exported mass spectra was performed in MassyTools using the same calibrants as listed in Supplementary Table 6. Calibration criteria were i) minimum S/N of nine for calibrants and ii) at least five calibrants per mass spectrum. Subsequently, the software performed a dynamic background subtraction followed by targeted data extraction for all analytes on the glycan composition list. Several quality parameters to evaluate the spectral quality (fraction of analytes above S/N 6 and absolute intensity) as well as analyte quality (S/N and an isotopic pattern based quality score [QC]) were exported along with the quantitative data (background subtracted analyte area's). Massy Tools output was further processed in Microsoft Excel 2010. Mass spectra were only included for statistical analyses if: i) calibration criteria were fulfilled, ii) the fraction of analytes above S/N 6 was more than 80%, and iii) if intensity of the spectrum was above 1.0×10^5 arbitrary units. Analytes were selected for statistical evaluation if in more than 20% of the included mass spectra for cases or controls (either in 20% of the included case spectra or in 20% of the included control spectra) their S/N was above 6 and QC was below 0.18. Replicate mass spectra were aggregated by averaging the background subtracted analyte areas. The last step before statistical analyses was normalization of the analyte areas to 100%.

Data analyses

Colorectal cancer related glycan alterations

Normalized data was imported into SIMCA software V13 (Umetrics, Umea, Sweden) and explored for potential batch effects and confounders such as plate, gender, age, and year of sampling (YOS) using a cross validated principal component analyses (PCA) with unit variance (UV) scaling. For cross-validation, random distribution of the data in 7 groups was used. Same data and parameters were used for supervised partial least squares discriminant analysis (PLS-DA) to reveal colorectal cancer related differences in serum *N*-glycosylation. Individual

glycans were evaluated by their variable importance in the projection (VIP). A VIP-value larger than one was considered significant. To confirm observed trends in PCA and PLS-DA and to allow for biologic interpretation, univariate analyses of the individual glycans were used as complementary approach. Because glycan intensities were not normally distributed, univariate differences were tested for significance using the Mann–Whitney *U* test. Qualitative differences, the relative up- or down-regulation of glycans in colorectal cancer, were used to further facilitate interpretation of serum glycobiology in CRC. Univariate analyses were performed in SPSS (Version 20, IBM, NY, USA).

CRC associated glycan alterations were assessed by several complementary approaches. The accordance in significant results between univariate analyses and PLS-DA derived VIP-values in the discovery set was explored. Subsequent evaluation consisted of univariate analyses in the independent validation set. Colorectal cancer related glycan alterations were considered validated (i.e. not the result of coincidental findings due to multiple testing) if PLS-DA VIP-value in the discovery set was >1, and if univariate *p*-value was <0.01 in both the discovery and validation set. The third evaluation consisted of paired univariate analyses in the postoperative validation set using the Wilcoxon Signed Ranks test. In the latter comparison, preoperative sera from the cases in the validation set were compared to postoperative sera from the same individuals, but now with a cancer free status. This postoperative validation was also used to rule out that the findings were biased because of the difference in freezer temperature between case and control storage for a short period of time. Differences were considered postoperatively validated if $P < 0.1$. And last, concordance of the found CRC associated qualitative differences (the relative up or downregulation in CRC) among the discovery set and the two validation sets was evaluated.

TSNG derived discriminant score

To evaluate the diagnostic potential of TSNG profiling in colorectal cancer, logistic ridge regression was used to calibrate a discriminant rule in the discovery dataset. Ridge regularization imposes a penalization over the maximum likelihood estimates of the regression coefficients so that they are shrunk towards zero. This allows to deal with a large number of highly correlated predictors. The amount of shrinkage is controlled by a penalization parameter which is proportional to the sum of squares of regression coefficients. The optimal value of the penalty parameter was obtained by 10-fold cross-validation. By applying the resulting estimated coefficients to the relative glycan intensities, case discriminant scores were generated for all study samples. The discriminative performance of this score was evaluated internally after double 10-fold cross-validation in the discovery set and externally in both validation sets by ROC curves.

Prognostic capacity of colorectal cancer related glycan alterations

Survival analyses were performed on the cohort of all cases to investigate which validated CRC-related glycan alterations were associated with prognosis. First, validated glycans and derived traits associated with CRC were dichotomized on every tenth percentile. For each created dichotomous variable, survival for the resulting two groups was estimated using the Kaplan-Meier method and compared with the log rank test. Subsequently, the optimal cut-off was determined as the dichotomization providing the largest separation. If survival differed significantly in univariate analyses, Cox regression modelling was used to adjust for age and stage.

TSNG derived prognostic score

Cox regression with ridge penalization was used for the calibration of a prognostic score based on relative glycan intensities on the curatively treated cohort. Solely the curatively treated patients were used for the calibration to show potential relevance for adjuvant treatment decision making. Age and stage were also included in the model as fixed effects and the linear predictor is proposed as prognostic score. Double 10-fold cross-validation was used to guarantee internal validation, i.e., we added an outer cross-validation layer and we repeated the calibration procedure ten times setting aside one part of the data and then applying the chosen optimized models to the left-out data. Analogously to the classification setting, performance of the resulting double cross-validated prognostic score was evaluated by time-dependent ROC curves [6], constructed for follow-up time points 1, 5 and 10 years. Additionally, the Kaplan-Meier method was used to estimate survival for two groups created by dichotomizing the double-cross validated prognostic score on the median. To compare the prognostic capacity of TSNG profiling to current clinical prognostication, a second prognostic rule was generated, but now solely based on the variables age and stage. The statistical significance of differences in AUCs at 1, 5 and 10 years (between the clinical prognostic score and the TSNG prognostic score) was assessed by 95% bootstrap confidence intervals.

REFERENCES

1. Ruhaak LR, Huhn C, Waterreus WJ, de Boer AR, Neusüss C, Hokke CH, Deelder AM, Wuhrer M. Hydrophilic interaction chromatography-based high-throughput sample preparation method for N-glycan analysis from total human

plasma glycoproteins. Anal Chem. 2008; 80:6119–26. https://doi.org/10.1021/ac800630x.

2. Reiding KR, Blank D, Kuijper DM, Deelder AM, Wuhrer M. High-throughput profiling of protein N-glycosylation by MALDI-TOF-MS employing linkage-specific sialic acid esterification. Anal Chem. 2014; 86:5784–93. https://doi.org/10.1021/ac500335t3.
3. Selman MH, Hemayatkar M, Deelder AM, Wuhrer M. Cotton HILIC SPE microtips for microscale purification and enrichment of glycans and glycopeptides. Anal Chem. 2011; 83:2492–99. https://doi.org/10.1021/ac10271165.
4. Ceroni A, Maass K, Geyer H, Geyer R, Dell A, Haslam SM. GlycoWorkbench: a tool for the computer-assisted annotation of mass spectra of glycans. J Proteome Res. 2008; 7:1650–59. https://doi.org/10.1021/pr70082520.
5. Jansen BC, Reiding KR, Bondt A, Hipgrave Ederveen AL, Palmblad M, Falck D, Wuhrer M. MassyTools: A High-Throughput Targeted Data Processing Tool for Relative Quantitation and Quality Control Developed for Glycomic and Glycoproteomic MALDI-MS. J Proteome Res. 2015; 14:5088–98. https://doi.org/10.1021/acs.jproteome.5b006589.
6. Heagerty PJ, Zheng Y. Survival model predictive accuracy and ROC curves. Biometrics. 2005; 61:92–105. https://doi.org/10.1111/j.0006-341X.2005.030814.x2.

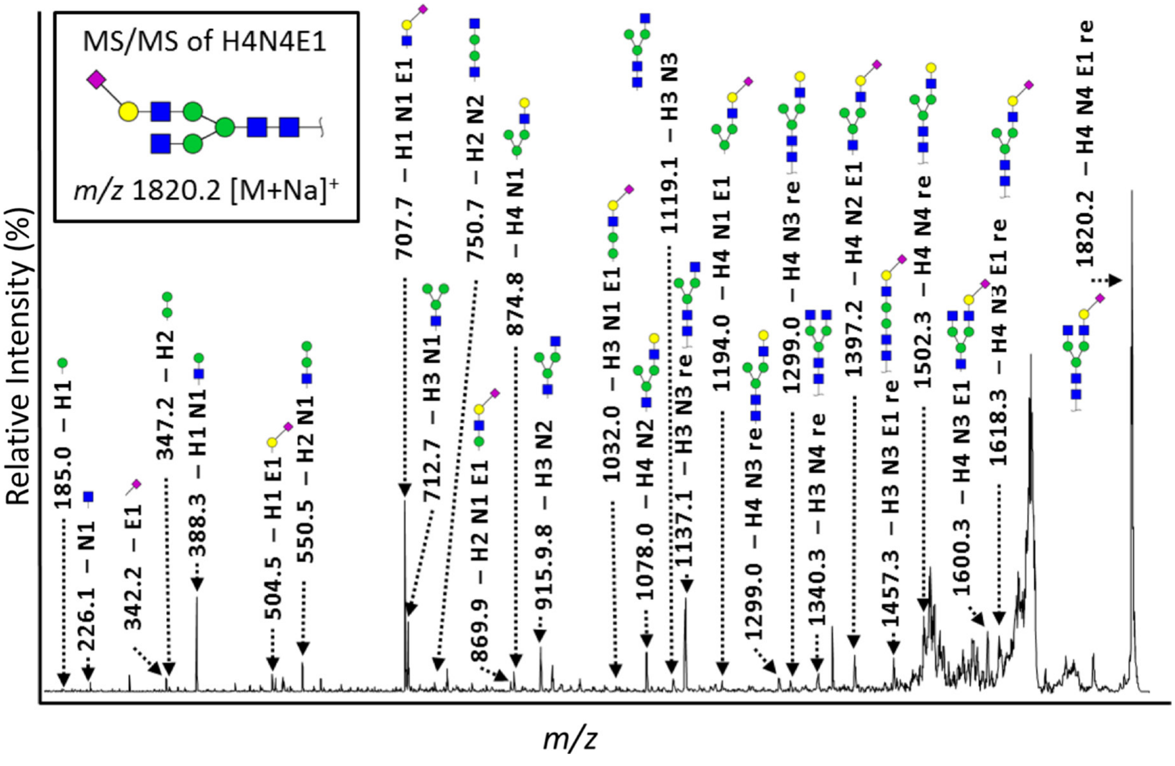

Supplementary Figure 1: Exemplary MALDI-TOF-MS/MS spectrum. Fragmentation of the peak at *m/z* 1820.2 confirms the glycan composition H4N4E1 with indicative fragment ions for the presence of α2,6-sialylation, though the presence of structural isomers cannot be excluded. Annotation was performed using GlycoWorkbench 2.1 stable build 146 (http://relax.organ.su.se/eurocarb/).

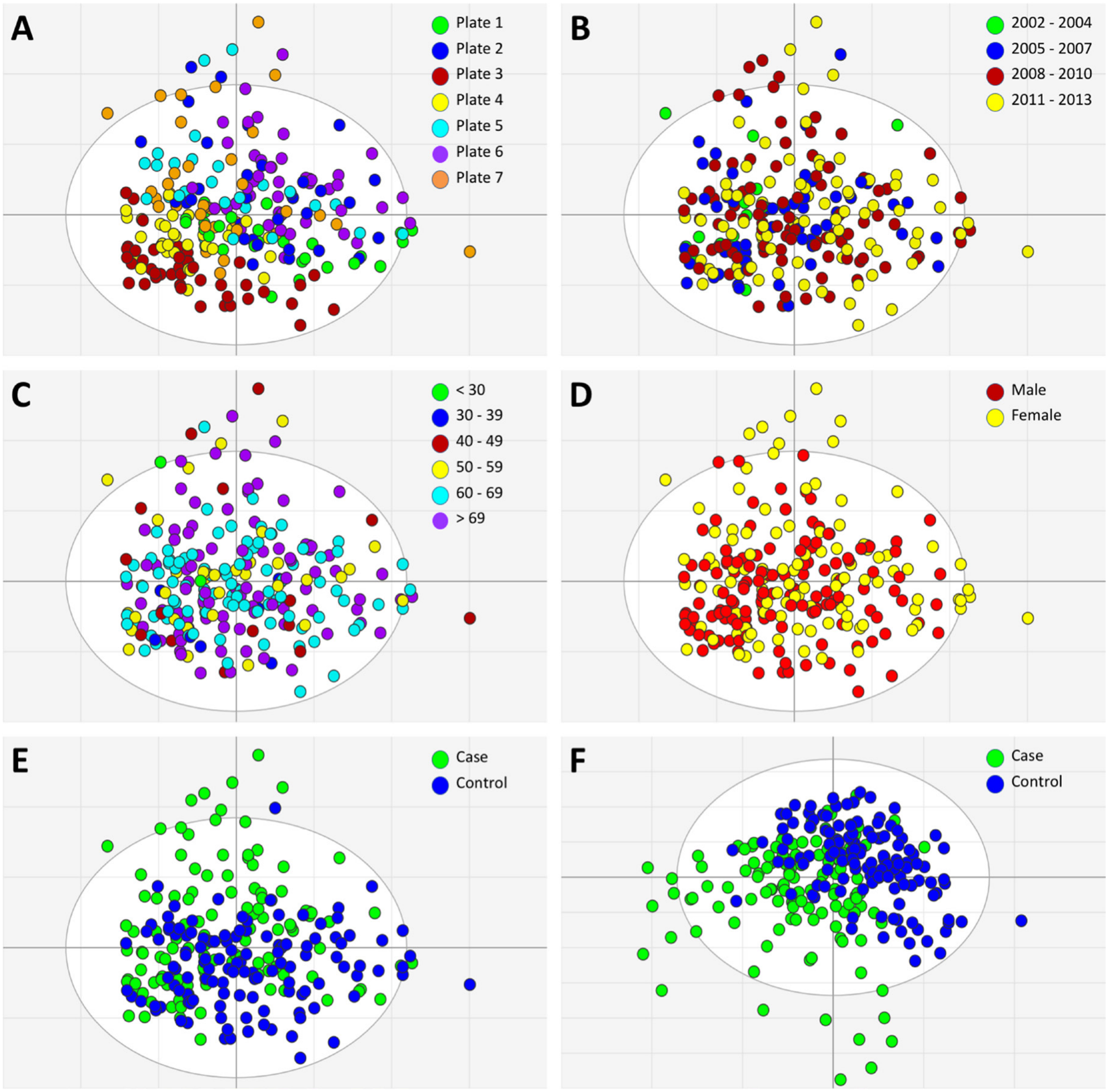

Supplementary Figure 2: Multivariate data analysis. Score plot of a cross validated principal component model fitted to the discovery dataset showing the first two components of twelve, colored to plate (**A**), year of serum sampling (**B**), age (**C**), sex (**D**) and case/control status (**E**). A clear clustering can be seen according to plate and case-control status. In the score plot of the cross validated partial least squares discriminant analysis (also fitted to the discovery data), separation is more clearly visible between cases and controls, but remains suboptimal (**F**).

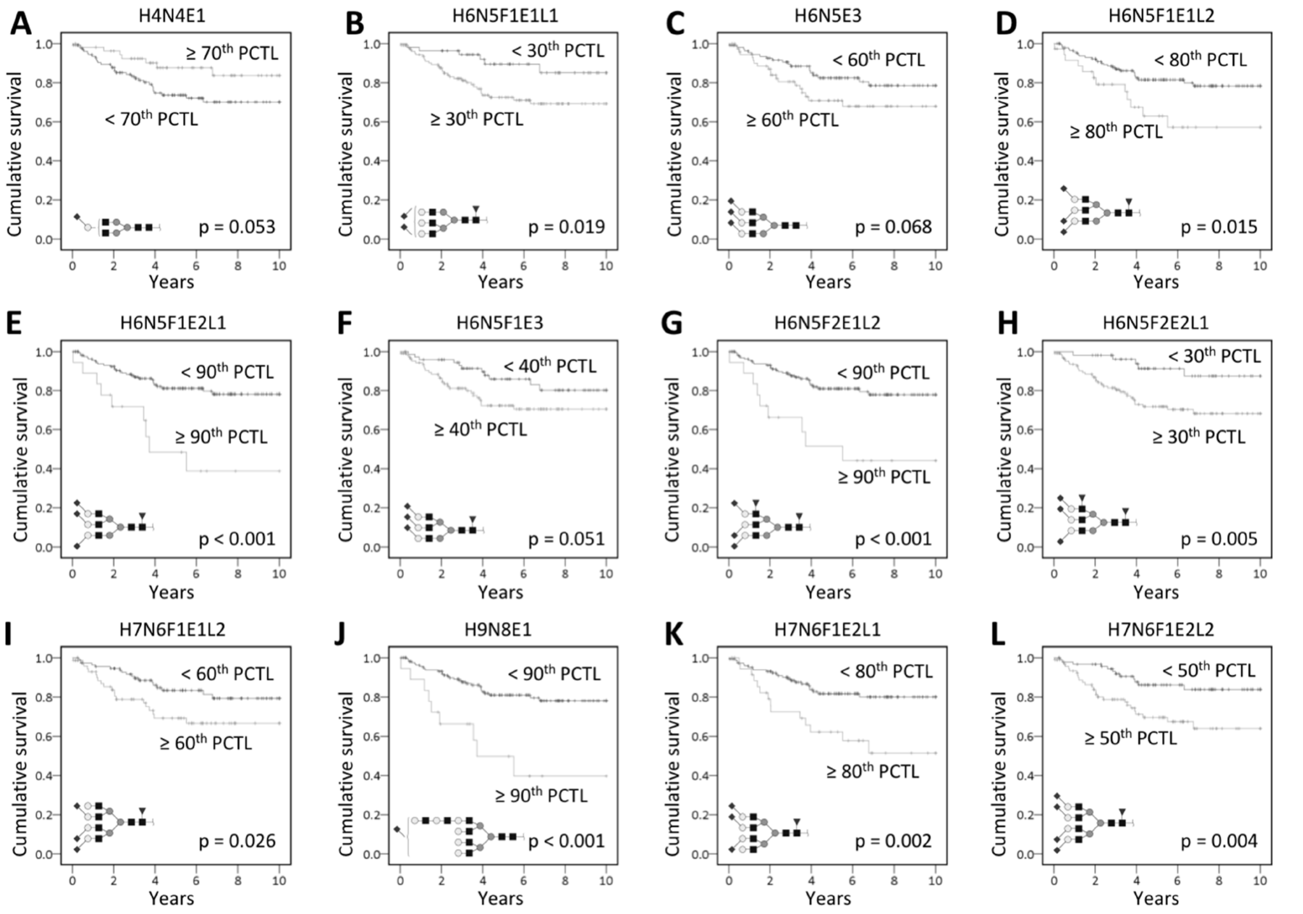

Supplementary Figure 3: Survival analyses. Survival analyses were performed on validated, single *N*-glycans with altered expression in CRC vs. controls (Table 2). Kaplan-Meier plots are shown for the *N*-glycans with prognostic potential, from which one was down-regulated (**A**) and eleven up-regulated (**B**–**L**) in CRC. PCTL, percentile; H = hexose; N = *N*-acetylhexosamine; E = (α2,6- linked *N*-acetylneuraminic acid; L = α2,3-linked *N*-acetylneuraminic acid; F = fucose.

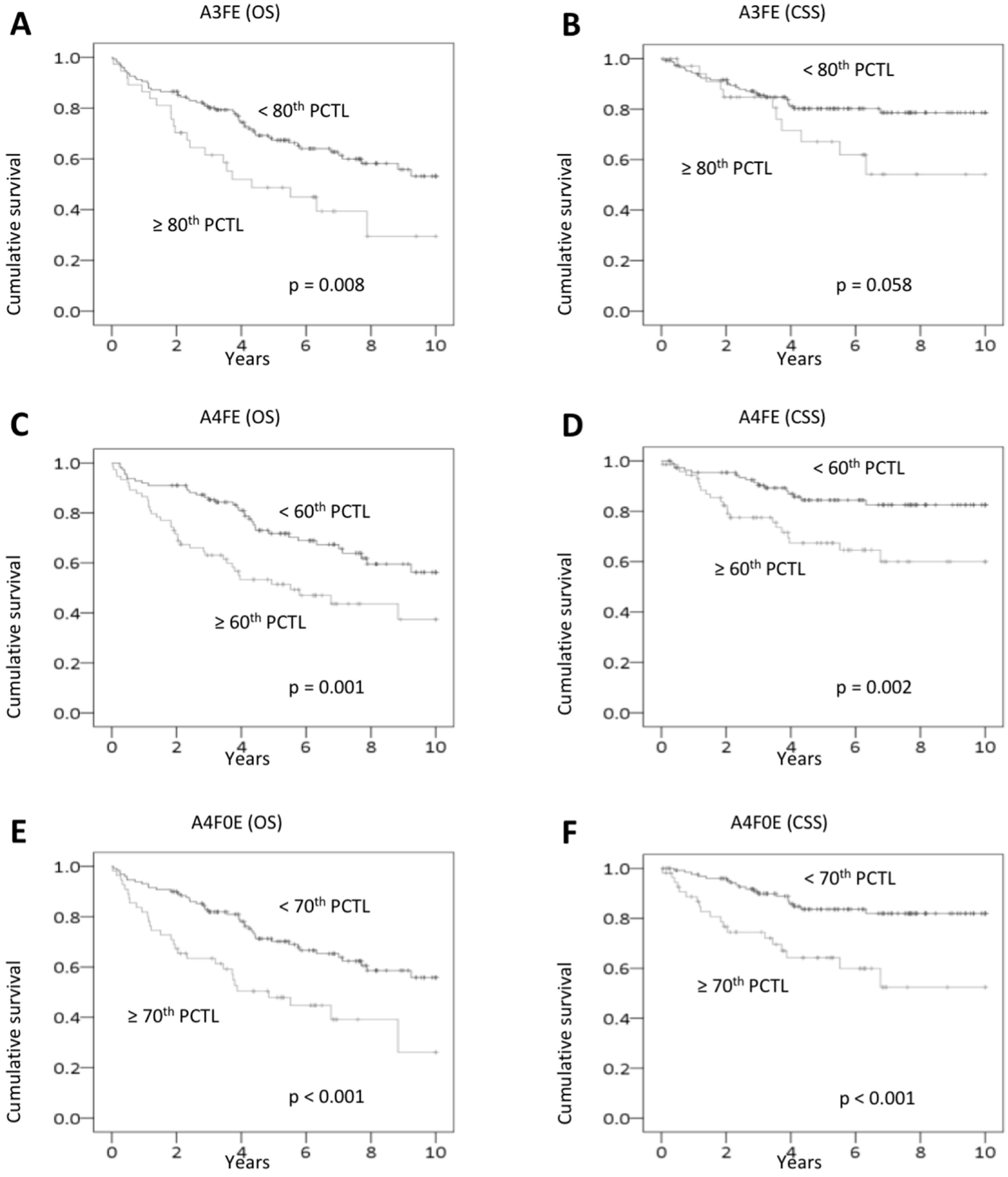

Supplementary Figure 4: Cancer specific survival. Kaplan–Meier plots representing the probability of cancer specific survival stratified by up or down-regulation of the concerning prognostic glycan with prognostic potential (**A**–**F**). PCTL, percentile; H = hexose; N = *N*-acetylhexosamine; E = (α2,6- linked *N*-acetylneuraminic acid; L = α2,3-linked *N*-acetylneuraminic acid; F = fucose.

Supplementary Table 1A–1G: Derived trait calculations. See Supplementary_Table_1

Supplementary Table 2: Peaklist and fragmentation information. See Supplementary_Table_2

Supplementary Table 3: Serum N-glycan alterations in colorectal cancer. See Supplementary_Table_3

Supplementary Table 4: Derived glycan trait alterations in colorectal cancer. See Supplementary_Table_4

Supplementary Table 5: Glycans associated with survival in colorectal cancer. See Supplementary_Table_5

Supplementary Table 6: Glycans used for internal calibration

Composition	m/z [M+Na]+
H3N4F1	1485.5337
H4N4F1	1647.5865
H5N4E1	1982.7081
H5N4E2	2301.8348
H5N4F1E2	2447.8927
H6N5E2L1	2940.0519
H6N5F1E2L1	3086.1098

m/z, mass to charge ratio; M, mass; Na, sodium; H, hexose; N, *N*-acetylhexosamine; F, fucose; E, α2,6-linked sialic acid; L, α2,3-linked sialic acid.

Supplementary Table 7: Glycans with prognostic potential in colorectal cancer

N-Glycan						Overall survival (77 events)						Cancer specific survival (38 events)					
						Univariate		Multivariate				Univariate		Multivariate			
Composition	Structure	*m/z* [M+Na]+	Alteration in CRC	Alteration with bad prognosis	Cut-off percentile			Adjusted for age		Adjusted for age and stage				adjusted for age		adjusted for age and stage	
						HR (CI)	*p*-value	HR (CI)	*p*-value	HR (CI)	*p*-value	HR (CI)	*p*-value	HR (CI)	*p*-value	HR (CI)	*p*-value
H4N4E1	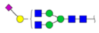	1820.66	down	down	0.7	0.5 (0.3–0.9)	0.018	0.5 (0.3–0.8)	0.007	0.5 (0.3–0.9)	0.014	0.5 (0.2–1.1)	0.060	0.4 (0.2–1.0)	0.051	0.4 (0.2–1.0)	0.049
H6N5F1E1L1	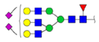	2766.98	up	up	0.3	2.2 (1.2–3.8)	0.007	2.0 (1.2–3.6)	0.014	1.8 (1.0–3.2)	0.037	2.7 (1.1–6.5)	0.024	2.7 (1.1–6.5)	0.026	2.3 (0.9–5.5)	0.068
H6N5E3	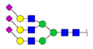	2986.09	up	up	0.6	1.8 (1.1–2.8)	0.011	1.7 (1.1–2.7)	0.026	1.5 (0.9–2.3)	0.098	1.8 (0.9–3.4)	0.072	1.8 (0.9–3.4)	0.083	1.4 (0.7–2.6)	0.352
H6N5F1E1L2	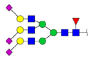	3040.07	up	up	0.8	2.1 (1.3–3.4)	0.004	2.2 (1.3–3.5)	0.003	1.7 (1.0–2.8)	0.040	2.3 (1.2–4.6)	0.018	2.3 (1.2–4.6)	0.017	1.6 (0.8–3.1)	0.211
H6N5F1E2L1	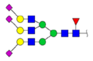	3086.11	up	up	0.9	2.3 (1.2–4.3)	0.008	2.4 (1.3–4.4)	0.006	2.0 (1.1–3.7)	0.032	3.7 (1.7–7.8)	0.001	3.7 (1.8–7.9)	0.001	2.7 (1.3–5.9)	0.010
H6N5F1E3	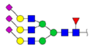	3132.15	up	up	0.4	2.2 (1.3–3.6)	0.002	2.1 (1.3–3.5)	0.004	1.8 (1.1–2.9)	0.029	2.0 (1.0–4.0)	0.056	2.0 (1.0–4.0)	0.062	1.6 (0.8–3.3)	0.180
H6N5F2E1L2	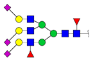	3186.13	up	up	0.9	2.1 (1.1–3.9)	0.017	2.2 (1.2–4.1)	0.012	1.9 (1.0–3.6)	0.042	3.5 (1.7–7.5)	0.001	3.6 (1.7–7.6)	0.001	2.7 (1.3–5.8)	0.010
H6N5F2E2L1	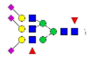	3232.17	up	up	0.3	2.1 (1.2–3.6)	0.011	2.1 (1.2–3.6)	0.010	1.9 (1.1–3.3)	0.027	3.5 (1.4–9.0)	0.009	3.45 (1.4–9.0)	0.009	3.3 (1.3–8.7)	0.014
H7N6F1E1L2	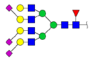	3405.20	up	up	0.6	1.8 (1.1–2.8)	0.011	1.7 (1.1–2.7)	0.023	1.3 (0.8–2.1)	0.265	2.0 (1.1–3.9)	0.029	2.0 (1.1–3.8)	0.033	1.3 (0.7–2.4)	0.473
H9N8E1	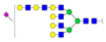	3443.24	up	up	0.9	2.2 (1.2–4.3)	0.014	2.1 (1.1–4.1)	0.021	1.7 (0.9–3.2)	0.132	3.8 (1.8–8.1)	0.001	3.8 (1.8–8.0)	0.001	2.6 (1.2–5.5)	0.015
H7N6F1E2L1	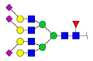	3451.24	up	up	0.8	2.1 (1.3–3.4)	0.003	1.9 (1.2–3.1)	0.009	1.7 (1.1–2.8)	0.026	2.7 (1.4–5.2)	0.003	2.7 (1.4–5.2)	0.004	3.0 (1.5–5.9)	0.002
H7N6F1E2L2	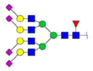	3724.33	up	up	0.5	2.0 (1.3–3.1)	0.004	1.9 (1.2–3.0)	0.008	1.6 (1.0–2.5)	0.055	2.6 (1.3–5.2)	0.006	2.6 (1.3–5.2)	0.007	2.1 (1.0–4.2)	0.043

m/z, mass to charge ratio; M, mass; Na, sodium; CRC, colorectal cancer; HR, hazard ratio; CI, 95% confidence interval; H, hexose; N, *N*-acetylhexosamine; F, fucose; E, α2,6-linked sialic acid; L, α2,3-linked sialic acid.
